# A new high-resolution melting analysis for the detection and identification of *Plasmodium* in human and *Anopheles* vectors of malaria

**DOI:** 10.1038/s41598-018-36515-9

**Published:** 2019-02-08

**Authors:** Enderson Murillo, Carlos Muskus, Luz A. Agudelo, Iván D. Vélez, Freddy Ruiz-Lopez

**Affiliations:** PECET, Program for the Study and Control of Tropical Diseases, Faculty of Medicine, University of Antioquia, Medellín, Colombia

## Abstract

Among vector-borne diseases malaria is the leading cause of morbidity in the world, with more than 200 million cases per year and a large number of deaths. The techniques traditionally used for the detection of *Plasmodium* in humans and *Anopheles* mosquitoes include microscopy, IRMA, ELISA, antibody or molecular assays, and anopheline dissection. However, these techniques are limited by their requirement of skilled personnel, low sensitivity or long processing times. A PCR-based high-resolution melting (PCR-HRM) analysis was developed for the detection and identification of *P*. *falciparum*, *P*. *vivax* and *P*. *malariae* that infect humans and *Anopheles*. In 41 human samples PCR-HRM detected 14 samples positive for *P*. *vivax*, 17 for *P*. *falciparum*, three for *P*. *malariae*, three mixed infections for *P*. *vivax*/*P*. *malariae* and four negative samples. Whereas benchmarking assays of microscopy and nested PCR had false positive detections. Additionally, PCR-HRM was able to detect natural infection with *Plasmodium* spp. in *An*. *darlingi* and *An*. *mattogrossensis*. The PCR-HRM presented is the first single assay developed for the detection and identification of *P*. *vivax*, *P*. *falciparum* and/or *P*. *malariae* in human and *Anopheles*. This method improves on currently available assays as it is easy-to-use, rapid, sensitive and specific with a low risk of contamination.

## Introduction

Among vector-borne diseases malaria is the main cause of morbidity in the world. It is estimated that in 2015 there were 212 million new cases and 429,000 mortalities due to malaria^1,2^. The main malaria species that infect humans are *Plasmodium vivax*, *P*. *falciparum*, *P*. *malariae* and *P*. *ovale*, of which, the first two, represent 95% of infections^1,3^. *Plasmodium* *malariae* has been reported in a limited number of cases in Colombia^4^, *P*. *ovale* predominates in Sub-Saharan Africa^5^ and recent studies have shown that *P*. *knowlesi*, *P*. *brasilianum* and *P*. *simiun* cause malaria in some human populations^6–8^. In Colombia in 2016, 83,356 malaria cases were reported, of which 33,055 (39.7%) were from *P*. *vivax*, 47,497 (57.0%) from *P*. *falciparum* and 2,804 (3.3%) from mixed malaria infections^9^.

Mosquitoes belonging to the genus *Anopheles* are the vectors of malaria in humans. This genus comprises approximately 465 species, of which it is estimated that 41 have epidemiological importance in malaria transmission^10^. Traditionally one of the most widely used methods to detect *Plasmodium* infection in the *Anopheles* has been dissection of the midgut and salivary glands to detect sporozoites under a microscope^11^. Subsequently, new techniques for the detection of natural infections in anophelines began to emerge, including immunoradiometric assays (IRMA)^12,13^ and the detection of the CS protein (of the circumsporozoite) by enzyme-linked immunosorbent assays (ELISA)^14^. However, these methods are time-consuming, require highly skilled personnel and have low sensitivity.

The diagnosis of malaria in humans is traditionally based on the microscopic detection of *Plasmodium* parasites in blood smears. This method is considered the gold standard diagnostic test for human malaria and is the most commonly used due to its low cost and simplicity^15^. However, disadvantages to this test include the requirement of skilled personnel, high levels of parasitemia (between 10–30 parasites/µl) and long processing times. In recent decades, as alternatives to microscopy, antibody (rapid tests) or molecular detection assays were introduced, but these also require long processing times and lack reliability in distinguishing between *Plasmodium* spp^16^. Furthermore, antibody assays have only been developed for the detection of antibodies against the antigens (HRP-2/pLDH) of *P*. *falciparum* and *P*. *vivax* and can lack sensitivity when parasitemia levels are low (<100 parasites/µl)^17–23^. Recently an ultra-sensitive *P*. *falciparum* HR2-based rapid diagnostic test was developed, which can detect at least three parasites/µl, however its accuracy may vary when there are deletions due to the different *P*. *falciparum* strains^24^.

Since the end of the 1980s molecular techniques based on the polymerase chain reaction (PCR) have been used in the detection of malaria in both humans and *Anopheles* due to their high sensitivity and efficient turnaround times in comparison to some traditional methods^15,16,25^. For example, Vernick *et al*.^26^ developed a reverse transcription-PCR assay to identify mosquitoes infected with *Plasmodium* spp. However, this assay was inefficient in the detection of *P*. *vivax* DNA in anophelines^26,27^. In a comparative manner with ELISA, nested PCR has also been used to detect and identify naturally and experimentally infected mosquitoes by using *Plasmodium*-specific genotype primers in a first round of amplification followed by species-specific primers in a second round of amplification^25,27,28^. Some disadvantages of nested PCR include the requirement of several rounds of PCR amplification to determine the species of *Plasmodium*^16^, the risk of contamination and extended assay processing times.

*Cytochrome b* (*Cytb*) and 18S ribosomal RNA (rRNA) are among the genetic targets commonly used for the molecular detection of *Plasmodium* that infect humans and *Anopheles* vectors. The emergence of real-time PCR for parasite detection and/or identification in humans and anophelines has improved the diagnosis of malaria by offering greater sensitivity (as few as 5 parasites/μl of blood required)^29–33^, easy processing with a lower risk of contamination. Currently, two detection systems have been used in such real-time PCR assays, the first based on the use of intercalators that bind to double-stranded DNA and the second based on the application of hybridisation probes. For example, Oddoux *et al*.^34^ developed a real-time PCR to detect up to five species of *Plasmodium* spp., however, this assay uses five species-specific primers to amplify *P*. *falciparum*, *P*. *vivax*, *P*. *malariae*, *P*. *ovale* and *P*. *knowlesi*, which is impractical from the point of view of cost and technical skill.

Intercalators are frequently used as they are cost-effective compared to hybridisation probes. Demonstrating the usefulness of intercalators, two methods have used these with good results. Chua *et al*.^35^ developed qRT-PCR-HRM for the simultaneous detection of *P*. *falciparum*, *P*. *knowlesi*, *P*. *malariae*, *P*. *ovale* and *P*. *vivax*. Also, Joste *et al*.^36^ developed a qPCR-HRM for detection of *P*. *ovale wallikeri* and *P*. *ovale curtisi*, with excellent results.

In this research we designed primers to target *Plasmodium* 18S rRNA^37,38^. This genomic target has six scattered copies across the *Plasmodium* genome, allowing the potential for greater assay sensitivity than targeting single copy genes^39^. Using a single set of primers and a high-resolution intercalating agent we then developed a new PCR-HRM assay for the detection and identification of the three main *Plasmodium* species that naturally infect humans and *Anopheles* in Colombia: *P*. *falciparum*, *P*. *vivax* and *P*. *malariae*.

## Results

### Design of primers for PCR-HRM

**Figure 1:**
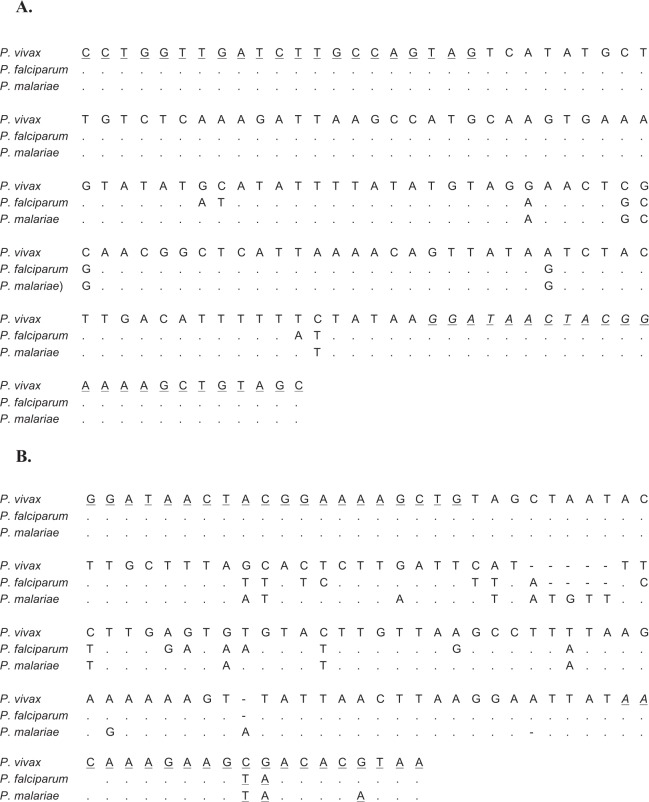
Alignment of nucleotide sequences of *P*. *vivax* (GenBank accession X13926), *P. falciparum* (M19172) and *P. malariae* (M54897). The primers flank the species-specific regions of *P*. *falciparum*, *P*. *malariae* and *P*. *vivax*. The nucleotides underlined correspond to the sense and antisense primers. (**A**) Primer pair Endmal18sF-R. (**B**) Primer pair Amzmal18sF-R.

Using the sequences reported for the 18S rRNA gene of *Plasmodium* spp. two pairs of primers were designed, Endmal18sF-R and Amzmal18sF-R (Fig. 1). The Endmal18sF-R primers (Fig. 1A) were designed to target single nucleotide polymorphisms (SNPs) unique to each *Plasmodium* spp., whereas Amzmal18sF-R targeted *Plasmodium* species-specific SNPs, as well as, size varying insertions/deletions (Fig. 1B). For primer Amzmal18sR three degenerate bases were incorporated in order to increase the amplification success of the three *Plasmodium* species.

The primers Endmal18sF-CCTGGTTGATCTTGCCAGTAG and Endmal18sR-GCTACAGCTTTTCCGTAGTTATCC amplified a 162 bp fragment, whereas the primer pair Amzmal18sF-GGATAACTACGGAAAAGCTG and Amzmal18sR TTAYGTGTYRCTTCTTTGTT amplicons varied in length from 132 to 137 bp, the length depending on the species of *Plasmodium* targeted (Fig. 1B). As both primer pairs amplified species-specific regions, the effect of these sequence variations were investigated to establish whether this allowed species discrimination through melting curve analyses.

### Theoretical estimation of the melting temperatures of the amplicons obtained for *P. vivax*, *P*. *falciparum* and *P*. *malariae*

The theoretical melting temperatures (T_m_) calculated with Oligonucleotide Properties Calculator (OligoCalc)^40^ for *P*. *vivax*, *P*. *falciparum* and *P*. *malariae* with the Endmal18sF-R primers were 78.3 °C, 77.6 °C and 78.2 °C, respectively. While with uMelt v2.0.2^41^ were 78.1 °C, 77.5 °C and 78.2 °C. For the Amzmal18sF-R primers the T_m_ were 76.2 °C, 73.9 °C and 73.7 °C with OligoCalc and 77 °C, 74 °C and 74.5 °C with uMelt, respectively (Supplementary Table S1).

### Optimal PCR annealing temperatures

Using conventional PCR the primers Endmal18sF-R and Amzmal18sF-R demonstrated a good ability to amplify DNA isolated from the positive control of *P*. *falciparum* 3D7. The Endmal18sF-R amplicons were generated from 45.4 °C to 60 °C, whereas the Amzmal18sF-R amplicons were generated from 45.4 °C to 53 °C. We determined the optimal annealing temperatures for the PCR-HRM assay as 55 °C or 52 °C for the Endmal18sF-R or Amzmal18sF-R primers, respectively.

### Assay specificity and sensitivity of PCR-HRM for *Plasmodium* spp

The standard curve in the PCR-HRM assay using the Endmal18sF-R and Amzmal18sF-R primers showed good correlation (0.99), with an efficiency of 93%. Our PCR-HRM assay achieved a good limit of detection to *Plasmodium* spp. parasitemia levels, where the amplification range was 10^1^ to 10^6^ copies (Supplementary Fig. S1). Additionally, it was determined that the ratio of 1:100 *P*. *falciparum*:interfering DNA did not inhibit the PCR-HRM reaction. However, an effect on the amplification of the PCR does occur, which is observed by a change in the Ct value. For both primer pairs the Ct value is higher in the presence of the interfering DNA (Supplementary Fig. S2).

Our PCR-HRM assay for both primer pairs showed specificity for *Plasmodium* DNA as there was no amplification with human DNA or *An*. *darlingi* DNA. We also conducted an *in silic*o specificity analysis demonstrating that the Endmal18sF-R and Amzmal18sF-R primers were non-specific to the genomes of human and *Anopheles* spp. Furthermore, no similarity was observed against the genomes of *Leishmania* spp., *Leptospira interrogans*, *Rickettsia* spp., *Brucella* spp., *Mycobacterium tuberculosis*, dengue serotype 1–4, Zika virus, Chikungunya virus and Yellow fever virus. Additionally, only partial similarity of the two primer pairs was observed against the DNA of *Toxoplasma gondii* and *Trypanosoma cruzi*.

**Figure 2:**
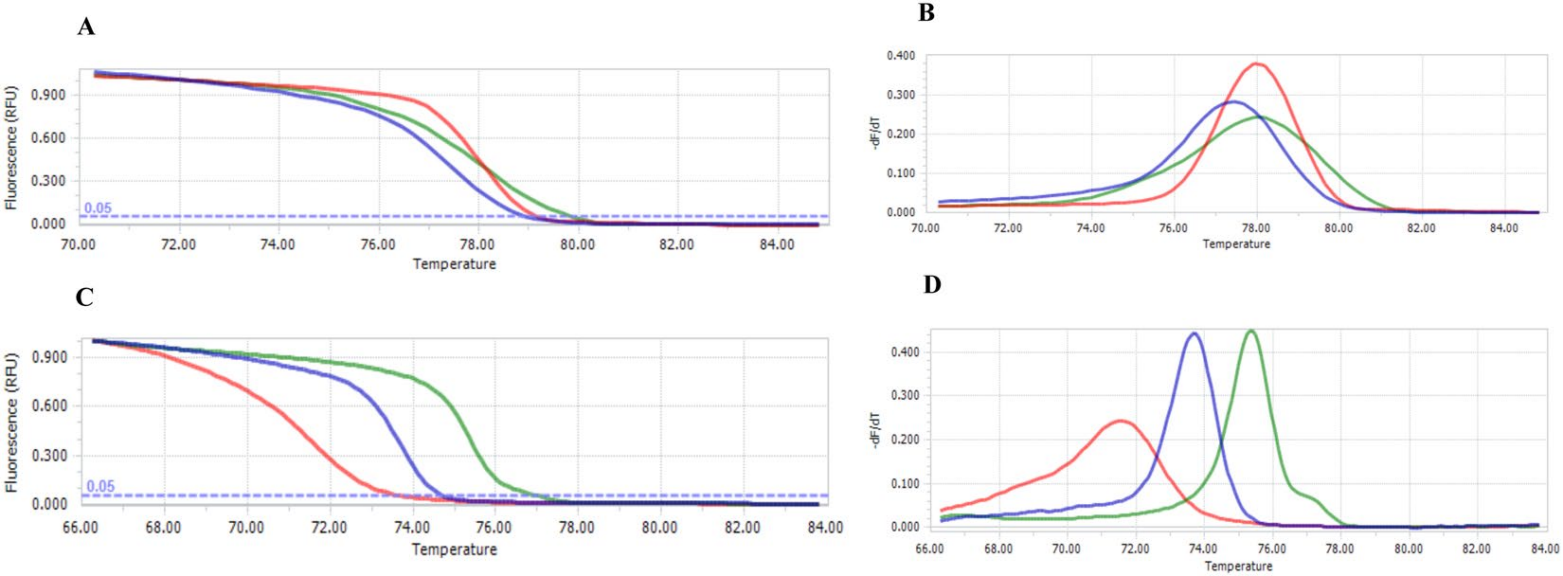
PCR-HRM analysis. Graphical representation of the melting curves. (**A,B**) Normalised melting curves and peaks using the Endmal18sF-R primers. (**C,D**) Normalised melting curves and peaks using the Amzmal18sF-R primers. Blue curve: *P. falciparum*. Green curve: *P*. *vivax*. Red curve: *P*. *malariae*.

The PCR-HRM assay using the primers Endmal18sF-R and Amzmal18sF-R succeeded in distinguishing the three species of *Plasmodium* studied. The melting curves were plotted as normalised melting curves and peaks using the LightCycler® 96 software v1.1.0.1320. The melting curves and experimental T_m_ showed a clear separation between the evaluated species, allowing each species to be determined unambiguously using the Endmal18sF-R (*p* < 0.001) and Amzmal18sF-R (*p* < 0.0001) primers (Table 1 and Fig. 2).

**Table 1:** Average melting temperatures (T_m_) in the PCR-HRM of the positives control of the three *Plasmodium* species.

*Plasmodium* species	T_m_ ± Sd (°C) Primer Endmal18sF-R	ANOVA	T_m_ ± Sd (°C) Primer Amzmal18sF-R	ANOVA
*Plasmodium falciparum*	77,38 ± 0,04	*p* < 0.0001*	73,69 ± 0,02	*p* ≪ 0.0001*
*Plasmodium vivax*	77,72 ± 0,02		75,50 ± 0,01	
*Plasmodium malariae*	78,55 ± 0,02		71,09 ± 0,01	

Sd: Standard deviation; T_m_: Melting Temperature; *ANOVA, *Significant difference among means (*p* < 0.05).

The experimental T_m_ obtained from the PCR-HRM analyses performed on the amplicons obtained with the Endmal18sF-R primers differentiated *P*. *vivax*, *P*. *falciparum* and *P*. *malariae* with a minimum difference around 0.38 °C between the three species. However, although a similar overall result using the Amzmal18sF-R primers was obtained they showed a more marked difference in the T_m_ between each of the three species. This allowed greater discrimination (greater difference in the T_m_) between *P*. *vivax* and *P*. *malariae* than the Endmal18sF-R primers (Table 1).

**Figure 3:**
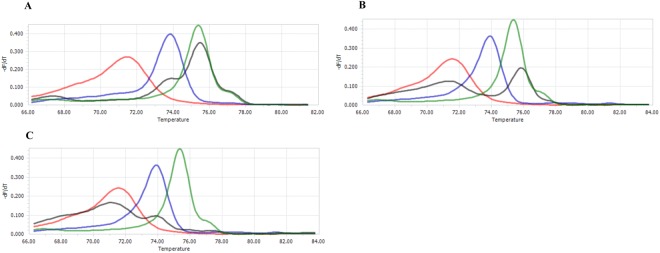
Melting curves for mixed malaria infection analysed by PCR-HRM. Mix infection with primers Amzmal18sF-R: (**A**) *P. falciparum*/*P*. *vivax*. (**B**) *P. malariae*/*P*. *vivax*. (**C**) *P. malariae*/*P*. *falciparum*. Black curves: Mixed infection. Red curve: *P. malariae*. Blue curve: *P. falciparum*. Green curve: *P. vivax*.

We also demonstrated that our PCR-HRM assay could experimentally distinguish a mixed infection (*P*. *vivax/P*. *falciparum*, *P*. *malariae*/*P*. *vivax* and *P. malariae*/*P*. *falciparum*) with the primers Amzmal18sF-R (Fig. 3). However, this distinction was not observed with the Endmal18sF-R primers as the melting curves and the T_m_ obtained were ambiguous showing similar values to a monoinfection.

### Preliminary validation of the PCR-HRM in human clinical and mosquito samples

In the 41 clinical samples of DNA extracted from human blood, PCR-HRM detected 14 samples positive for *P*. *vivax*, 17 for *P*. *falciparum*, three for *P*. *malariae*, three mixed infections for *P*. *vivax*/*P*. *malariae* and four negative samples. These results did not concur with two benchmarking assays. The first by counting *Plasmodium* parasites in blood smears under a microscope, which only observed 26 positive samples: ten *P*. *vivax*, 15 *P*. *falciparum* and one mixed infection of *P*. *vivax*/*P*. *malariae*. The second, nested PCR^16^ had 31 positive samples: 13 *P*. *vivax*, 15 *P*. *falciparum*, two *P*. *malariae* and one mixed infection of *P*. *vivax*/*P*. *malariae* (Table 2). Additionally, the Kappa index between blood smears and our PCR-HRM with the primers Endmal18sF-R was *k* = 0.373 IC 95% and Amzmal18sF-R was *k* = 0.549 IC 95%. Between our PCR-HRM and nested PCR we observed with the primers Amzmal18sF-R a Kappa index of *k* = 0.739 IC 95%, and with Endmal18sF-R was *k* = 0.396 IC 95%.

**Table 2:** Identification of *Plasmodium* spp. in human clinical and *Anopheles* vector samples by PCR-HRM.

	PCR-HRM					Snounou’s Nested PCR					Blood smears				
	Pv	Pf	Pm	Mx	Neg	Pv	Pf	Pm	Mx	Neg	Pv	Pf	Pm	Mx	Neg
Human	14	17	3	3	4	13	15	2	1	10	10	15	0	1	15
*An*. *darlingi*	1	1	0	0	98	0	0	0	0	100	—	—	—	—	—
*An*. *mattogrossensis*	8	0	0	0	0	0	0	0	0	8	—	—	—	—	—
*Anopheles* spp.	0	0	0	0	234	0	0	0	0	234	—	—	—	—	—

The Endmal18sF-R and Amzmal18sF-R primers were used to evaluate human samples (N = 41) and samples from *Anopheles* spp. (N = 342) collected in an area endemic with high malaria transmission in Amazonas, Colombia. PCR-HRM: Total detection using both primer pairs. Pv: *P. vivax*. Pf: *P. falciparum*. Pm: *P. malariae*. Mx: Mixed infection Pv/Pm only detected with Amzmal18sF-R primers. Neg: No detection.

Similarly, 342 anophelines captured from an endemic area with high malaria transmission, thus having a high suspicion of being infected naturally with *Plasmodium* spp., were evaluated. Our PCR-HRM assay with primers Endmal18sF-R and Amzmal18sF-R detected two *An*. *darlingi* positives with *P*. *vivax* and *P*. *falciparum*, and eight *An*. *mattogrossensis* positives with *P*. *vivax*. The remaining samples were negative (Table 2, Supplementary Figs S3, S4 and Supplementary Table S2). The positive PCR amplicons were confirmed by sequencing.

## Discussion

Three of the four *Plasmodium* spp. - *P*. *falciparum*, *P*. *vivax* and *P*. *malariae* - traditionally known to infect humans and causing malaria are present in Colombia. Commonly, the techniques used to detect and identify these species include microscopy or by PCR-based assays using genus - and/or species-specific primers^16,25,32–34^. Microscopy, although an economical and rapid technique that can be performed in rural areas with simply equipped hospitals or health centres, still requires an experienced microscopist and a high-level of infection in the sample; low or mixed parasitemia are not easy to detect leading to false negative tests. Typically, the detection and identification of *Plasmodium* spp. that infect humans by PCR requires the running of two independent reactions, which increases costs and extends test turnaround times. We present here the results of the implementation of a PCR-HRM assay that allows the rapid and accurate detection and identification of three *Plasmodium* spp., in either clinical samples or *Anopheles* vector samples. Our assay differs from those PCR-HRM already published^35,36,42^ that have only been used to detect *Plasmodium* spp. in clinical samples.

The PCR-HRM assay developed was successful in detecting and identifying three of the main species of *Plasmodium*, with two sets of independent primer pairs Endmal18sF-R and Amzmal18sF-R. The Amzmal18sF-R primer pair were more accurate than the Endmal18sF-R primers. This a consequence of the Amzmal18sF-R design incorporating both SNPs and insertions/deletions leading to amplicon length differences that gave more effective discrimination between each of the *Plasmodium* species.

The technique presented improved on established PCR assays that test for *Plasmodium* infection^15,16,25,26,28,34^ as it offers the ability to detect and discriminate between the three *Plasmodium* spp. using a single set of primers in a single round of PCR. An additional benefit of our assay using the Amzmal18sF-R primers was demonstrated by its sensitivity to detect and differentiate mixed infections of *P*. *falciparum*/*P*. *vivax*, *P*.*falciparum*/*P*. *malariae* and *P*. *vivax*/*P*. *malariae* (Fig. 3). This would be highly relevant in diagnostic healthcare provision as mixed infections of *P*. *vivax*/*P*. *falciparum* is the most predominant^1^. Further assay development is required to support the *in silico* predications that our PCR-HRM assay also has the potential to detect and discriminate between infections for *P*. *simium* and *P*. *brasilianum*, species genetically related with *P*. *vivax* and *P*. *malariae* (Supplementary Table S3). If experiments support this *in silico* prediction our PCR-HRM will improve *Plasmodium* testing as current PCR-HRM cannot discriminate between *P*. *simium* and *P*. *brasilianum*^25,26^.

We demonstrate that estimating the theoretical T_m_ is a good gauge to predict whether a PCR-HRM primer pair will be species-specific. Although the absolute values of the theoretical T_m_ differed from those obtained experimentally with both our primer pairs, the difference between the values was consistent in magnitude and allowed species discrimination.

It is important in diagnostics that any new test has equivalent sensitivity to established assays. We demonstrated that our PCR-HRM assay developed with both primer pairs had excellent sensitivity, being able to detect one parasite. This is an improvement on the sensitivity of microscopy that at best has a minimum sensitivity of 10–30 parasites/µl in blood^15,16^. Our PCR-HRM is also comparable to the nested PCR method of Snounou *et al*.^16^ - a benchmarking test for mosquito vector incrimination - and other established PCR-based assays^29,33,43^, having a limit of detection of at least one parasite (equivalent to 6 copies/μl). Eight samples of *An*. *mattogrossensis* and two of *An*. *darlingi* were negative by Snounou’s PCR^16^. However, with the PCR-HRM developed in this research these samples were positive for *Plasmodium* (Table 2), confirming the high sensitivity of our assay.

Our PCR-HRM was found to be within the expected range of efficiency of between 90–110%^44^. This allowed us to infer that amplicon generation with both primer pairs occurred without contamination. Although our assay is not as cost efficient as those established tests such as microscopy, ELISA and other PCR-based methods, it does present a number of advantages. Our assay is able to detect and identify *Plasmodium* spp. in a single round of PCR, which is not possible with the method of Snounou *et al*.^16,25^, and it minimises the presence of false positives as commonly encountered when testing by ELISA.

To the best of our knowledge, we report the first natural infection of *P. vivax* in *An*. *mattogrossensis* in Colombia. This anopheline has been found to be positive for *P*. *vivax* in Brazil and Peru^45,46^. We collected *An*. *mattogrossensis* in San Pedro de Tipisca and Doce de Octubre, Puerto Nariño, Amazonas. These localities have a high transmission of malaria throughout the year. We therefore purport that this species together with *An*. *darlingi* play a role in the transmission of malaria in these communities, which current mosquito programs do not recognise.

In conclusion, the PCR-HRM developed in this research is the first test developed in Colombia that can be used for the detection and identification of three species of *Plasmodium* that infect both human and anopheline vectors. We suggest the preferential use of Amzmal18sF-R for the PCR-HRM, as this primer pair can be used to detect and identify *P*. *vivax*, *P*. *falciparum*, *P*. *malariae* and mixed infections. Additionally, this methodology has multiple advantages with respect to established methods such as nested PCR^16^. Our PCR-HRM is easy-to-use, rapid, sensitive and specific, uses a single PCR reaction and has a low risk of contamination. Finally, this research is currently aimed at epidemiology-based research centres, but has the potential for diagnostic development and approval.

## Methods

The clinical samples (N = 41) and *Anopheles* mosquitoes (N = 342) were collected in the municipality of Puerto Nariño, communities of San Juan de Tipisca and Doce de October, during 2015 to 2016 as part of the project “Implementation of an Early Warning System for the Prevention and Control of the main ETV in the department of Amazonas, Colombia”. During this two year period the communities were visited in total eight times, once every three months. The clinical samples were obtained by active search of patients, or concentration of persons with malaria symptomatology or persons that belonged to malaria endemic areas. In parallel to human sample collection, *Anopheles* spp. were collected with CDC traps located overnight inside and outside of houses, for a minimum of two nights per location.

### Samples for PCR-HRM

Genomic parasite DNA of *P*. *vivax*, *P*. *falciparum* 3D7 and human *P*. *malariae* were supplied by the Malaria-SIU research laboratory of the University of Antioquia. For the clinical samples we used DNA extracted from blood using Qiagen® DNA extraction kit. For each individual we took two blood samples. Following the official malaria diagnostic protocol in Colombia, the first sample was used for blood smear^47^. The additional second sample was stored on FTA cards (Whatman®) for molecular techniques. For the mosquito malaria vector DNA, 342 samples of *Anopheles* spp. were captured from indigenous settlements in Puerto Nariño, Amazonas, Colombia, an area endemic with high malaria transmission. Subsequent to the morphological identification of the specimens, the head and thorax of each mosquito was separated from the abdomen. The *Plasmodium* DNA extraction was made by macerating the specimens (head-thorax) for 30–40 seconds in 20 µl of Milli-Q water. Then 20 µl of 10% Chelex ® was added and heated at 56 °C for 10 minutes followed by 99 °C for 10 minutes. Finally, the solution was centrifuged at 12,000 rpm for 5 minutes and the supernatant (DNA) was separated and stored at −20 °C. The established methods of *Plasmodium* counts from blood smear under a microcope^47^ and nested PCR described by Snounou *et al*.^16^ were used as benchmarking assays to establish the detection of *Plasmodium* spp.

### Genomic target selection and primer design for PCR-HRM

PCR-HRM primers were designed by targeting both a highly conserved and a variable region of 18S rRNA of *Plasmodium* spp. We used GenBank (NCBI, Bethesda MD, USA) sequence accession numbers M19172 for *P*. *falciparum*, M54897 for *P*. *malariae* and X13926 for *P*. *vivax*. Primer3Plus (http://www.bioinformatics.nl/cgi-bin/primer3plus/primer3plus.cgi) was used to design two pairs of primers, Endmal18sF-R and Amzmal18sF-R, to specifically target the three *Plasmodium* spp. The design quality of the oligonucleotides was evaluated by OligoAnalyzer v3.1 (https://www.idtDNA.com/calc/analyzer) to avoid homodimers and heterodimers. Primer specificity was analysed by Primer-BLAST (https://www.ncbi.nlm.nih.gov/tools/primer-blast/).

### *In silico* simulation of melting curves of *P*. *vivax*, *P*. *falciparum* and *P*. *malariae* for PCR-HRM

*In silico* simulation of the melting curves was based on the sequence region amplified by Endmal18sF-R and Amzmal18sF-R primers. The theoretical melting temperatures (T_m_) were calculated using OligoCalc (http://biotools.nubic.northwestern.edu/OligoCalc.html) and uMelt v2.0.2 (http://www.dna.utah.edu/umelt/um.php).

### Evaluation of the optimal primer annealing temperature

A PCR-based temperature gradient was created to ensure that the two pairs of primers developed were able to amplify the target region in the three *Plasmodium* species, avoiding non-specific amplification or primer dimers that interfere with the interpretation of the subsequent results in the PCR-HRM analysis.

Target sequences were amplified for each primer pair independently. Reaction conditions included: 12.5 μl PCR Master Mix 2X(Thermo Fisher Scientific), 1.25 μl of each primer (10 μM), 9 μl of DNase-free water and 1 μl of DNA (10–20 ng/μl) for a final volume of 25 μl per sample. Thermocycling conditions were one cycle of 95 °C × 5 minutes, followed by 35 cycles of denaturation at 95 °C × 15 seconds, annealing from 45 °C to 60 °C × 20 seconds, extension at 72 °C × 20 seconds and a final extension at 72 °C × 10 minutes. The SimpliAmp® thermal cycler (Applied Biosystems, CA, USA) was used and the products were visualised on a 2% agarose gel.

### PCR-HRM for *Plasmodium* spp

PCR-HRM was evaluated for *P*. *falciparum* DNA positive controls, human clinical samples or DNA isolated from *Anopheles* spp. All the samples were replicated twice. The reactions were prepared containing 6 μl PCR Master Mix 2X(PCR-HRM Qiagen, Hilden, Germany), 0.24 μl of each Endmal18sF-R primer (10 μM) (except 1 μl of each primer Amzmal18sF-R) and 2 μl of DNA (10–20 ng/μl) in a final volume of 12 μl made-up with DNA-free water (Qiagen, Hilden, Germany). Using a Roche LightCycler® 96, the reactions were amplified using one cycle of 95 °C for 5 minutes, followed by 50 cycles of 95 °C for 10 seconds, 55 °C for the Endmal18sF-R primers and 52 °C for the Amzmal18sF-R primers for 20 seconds and extension at 72 °C × 20 seconds. The PCR-HRM analysis was carried out from 70 °C to 85 °C with a step increase of 0.10 °C/second for the primer pair Endmal18sF-R and 0.25 °C/second for the primer pair Amzmal18sF-R. Finally, the fusion curves were generated and analysed using a RocheLightCycler® 96 v1.1.0.1320 program to determine the T_m_ for each species of *Plasmodium*. Samples were sequenced at the commercial company Macrogen (Korea) and are publically available at GenBank, accessions numbers MH614626-29.

### PCR-HRM assay sensitivity and specificity

To estimate the limit of detection of the PCR-HRM the amplicons derived from *P*. *falciparum* 3D7 using the primers Endmal18sF-R and Amzmal18sF-R were purified and cloned into the vector pGEM®-T Easy (Promega, Madison WI) according to the manufacturer’s specification. Serial ten-fold dilutions of a solution of the purified clone were made. The range of concentrations used was between 10^1^ to 10^6^ copies of the plasmid containing the amplified *P*. *falciparum*/μl. Each DNA concentration was assayed in duplicate by PCR-HRM.

To evaluate the specificity of the PCR-HRM, DNA from *An*. *darlingi* and from uninfected human DNA were used. The assay was performed using 50 ng of these DNAs per PCR-HRM reaction. We evaluated whether the presence of interfering DNA from *An*. *darlingi* and from humans could inhibit the PCR-HRM reaction. 10 ng of *P*. *falciparum* 3D7 was used in a 1:1 and 1:100 ratio with the interfering DNA.

*In silico* analysis was performed to evaluate the specificity of the assay against DNA of different microorganisms with febrile syndrome: *Toxoplasma gondii*, *Leishmania* spp., *Trypanosoma cruzi*, *Leptospira interrogans*, *Rickettsia* spp., *Brucella* spp., *Mycobacterium tuberculosis*, dengue serotype 1–4, Zika virus, Chikungunya virus and Yellow fever virus.

### Statistical analysis

We tested the presence of atypical value using the Tukey test. We analysed the data using GraphPad Prism v7.04. The diagnostic of the concordance of the clinical samples was calculated using the Kappa index between blood smear, nested PCR and PCR-HRM. We also calculated summary measures using ANOVA with a statistical significance of *p* < 0.05.

### Ethics approval and consent to participate

This protocol was approved by the Human Experimentation Ethics Committee of the University of Antioquia. For all cases we obtained informed consent of the patients included in the study. The study protocol was carried out in accordance with the ethical standards of the Declaration of Helsinki 1975 and Resolution 008430 of 1993 of the Ministry of Health of Colombia.

## Electronic supplementary material

Supplementary Files

## Data Availability

Patient information are not public due to the confidential nature of the collected personal data, but are available from the corresponding author upon reasonable request.
