# Supplementary material for: A new high-resolution melting analysis for the detection and identification of Plasmodium in human and Anopheles vectors of malaria
Source: Sci Rep. 2019 Feb 8;9:1674. doi: 10.1038/s41598-018-36515-9 (PMC6368607; doi:10.1038/s41598-018-36515-9)
Supplement: Supplementary file 1 — Supplementary Files [file 41598_2018_36515_MOESM1_ESM.pdf]

A new high-resolution melting analysis for the detection and identification of *Plasmodium* in human and *Anopheles* vectors of malaria

Enderson Murillo, Carlos Muskus, Luz A. Agudelo, Iván D. Vélez, Freddy Ruiz-Lopez

Supplementary Table S1. Theoretical melting temperatures of the three species of *Plasmodium* studied. The theoretical melting temperatures (T_m) were calculated using OligoCalc (http://biotools.nubic.northwestern.edu/OligoCalc.html) and uMelt v2.0.2 (http://www.dna. utah.edu/umelt/um.php), for the three species studied: *P. vivax, P. falciparum* and *P malariae*. The GenBank accession numbers listed were used to simulation of theoretical temperature. The sequences were aligned in Mega 6. Only the sequences with the full amplicons were analysed.

***Plasmodium* species**	**GenBank accession**	**Endmal18SF-R theoretial T_m**		**GenBank accession**	**Amzmal18SF-R theoretial T_m**	
		**uMelt**	**OligoCalc**		**uMelt**	**OligoCalc**
P. vivax	X13926	79	79.05	X13926	77	76.15
P. vivax	LT635613	79	79.05	LT615241	77	76.15
P. vivax	LT615265	79	79.05	JQ627158	77	76.15
P. vivax	LT615257	79	79.05	U83877	77	76.15
P. vivax	LT615240	79	79.05	JQ627155	77	76.15
P. vivax	U07367	79	79.05	JQ627156	77	76.15
P. vivax	U03079	79	79.05	JQ627157	77	76.15
P. vivax	LT635621	79	79.05	JQ627153	77	76.15
P. falciparum	M19172	77.5	77.64	XR_002273101	74	73.91
P. falciparum	XR_002273101	77.5	77.64	XR_002273080	74	73.91
P. falciparum	XR_002273080	77.5	77.64	CP016997	74	73.91
P. falciparum	CP016997	77.5	77.64	CP016995	74	73.91
P. falciparum	CP016995	77.5	77.64	AL844506	74	73.91
P. falciparum	AL844506	77.5	77.64	AL844504	74	73.91
P. falciparum	AL844504	77.5	77.64	pdb|3J7A|A	74	73.91
P. falciparum	pdb|3J7A|A	77.5	77.64	JQ627151	74	73.91
P. malariae	M54897	78.2	78.22	AB489195	74.5	73.68
P. malariae	LT594631	78.2	78.22	AB489196	74.5	73.68
P. malariae	LT594624	78.2	78.22	LT594491	74.5	73.68
P. malariae	LT594624	78.2	78.22	---------	---------	---------
P. malariae	AF488000	78.2	78.22	---------	---------	---------
P. malariae	AF487999	78.2	78.22	---------	---------	---------

A **Efficiency = 93%, R^2 = 0,99, LDO= 1 copies/µl**

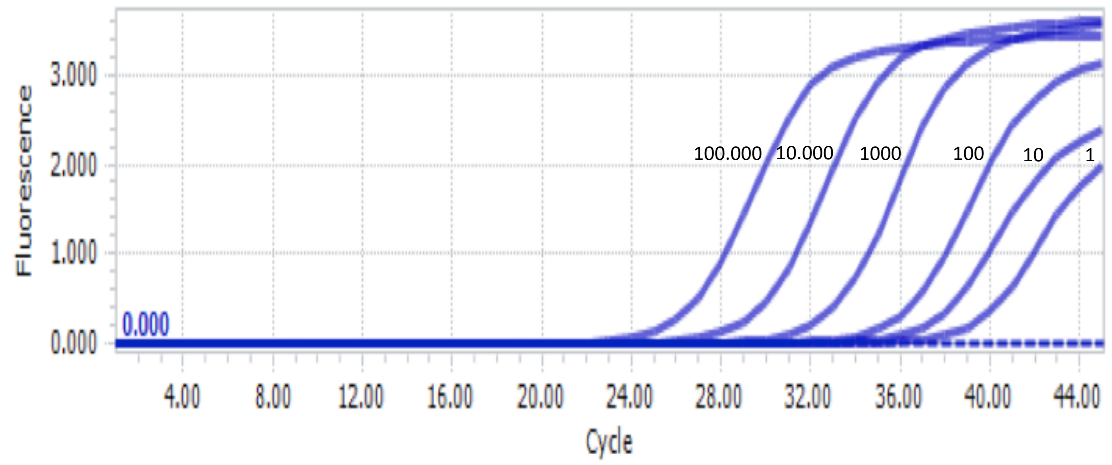

B **Efficiency = 93%, R^2 = 0,99, LDO= 1 copies/µl**

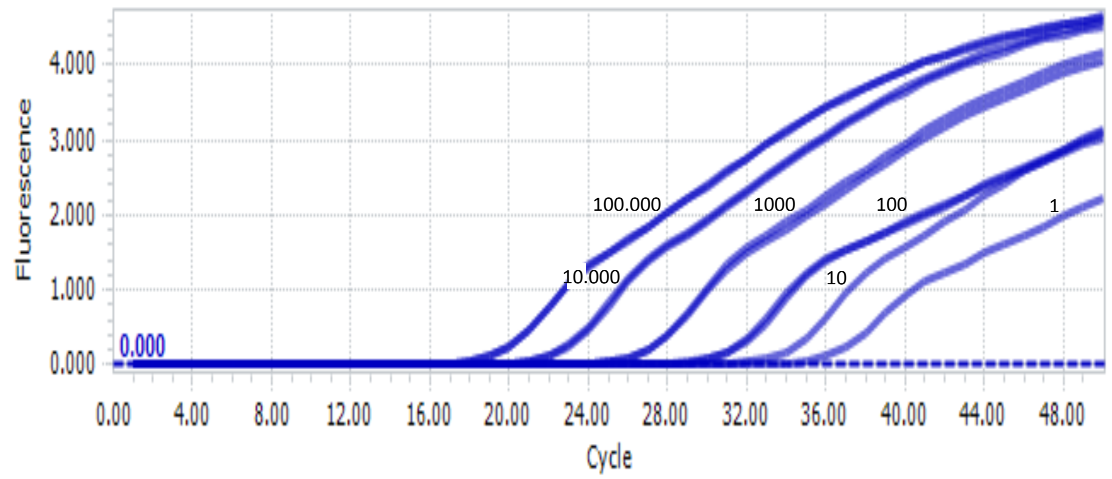

Supplementary Figure S1. PCR-HRM standard curves. Standard curve with six point of dilution on base 10, of a plasmid of *P. falciparum* 3D7, using LightCycler® 96 v1.1.0.1320. Primers: **A.** Endmal18SF-R. **B.** Amzmal18SF-R.

A

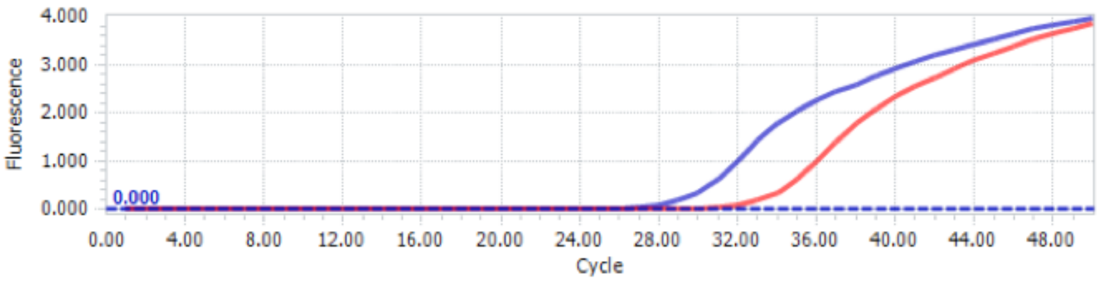

B

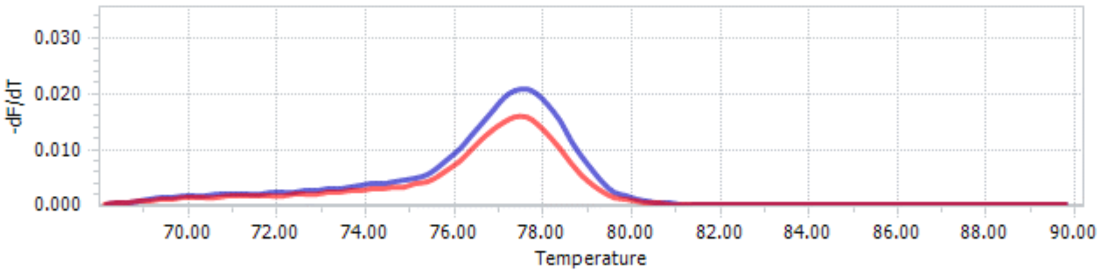

Supplementary Figure S2. DNA interfering Assay. For Endmal18sF-R primers: **A**. Amplification curve. **B.** Melting curve of *P. falciparum* 3D7. 10ng of *P. falciparum* 3D7 was used in a 1:1 (blue) and 1:100 ratio (red).

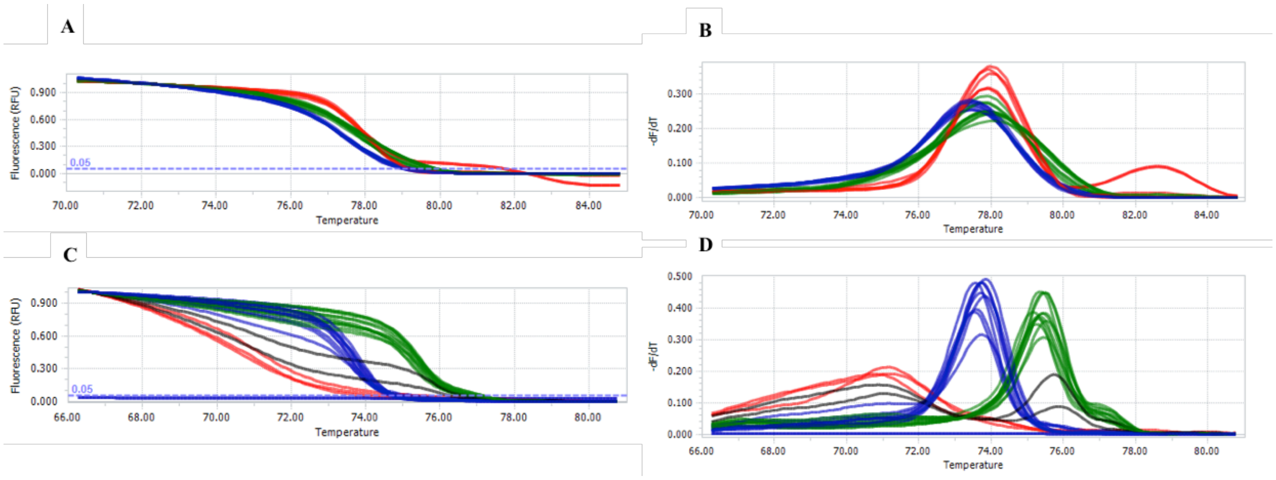

Supplementary Figure S3. PCR-HRM analysis of clinical samples. A and **B**. Normalised melting curves and peaks using the Endmal18sF-R primers. **C** and **D**. Normalised melting curves and peaks using the Amzmal18sF-R primers. Blue curve: *P. falciparum*. Green curve: *P. vivax*. Red curve: *P. malariae*. Black curves with the Amzmal18sF-R are mixed infection of clinical samples.

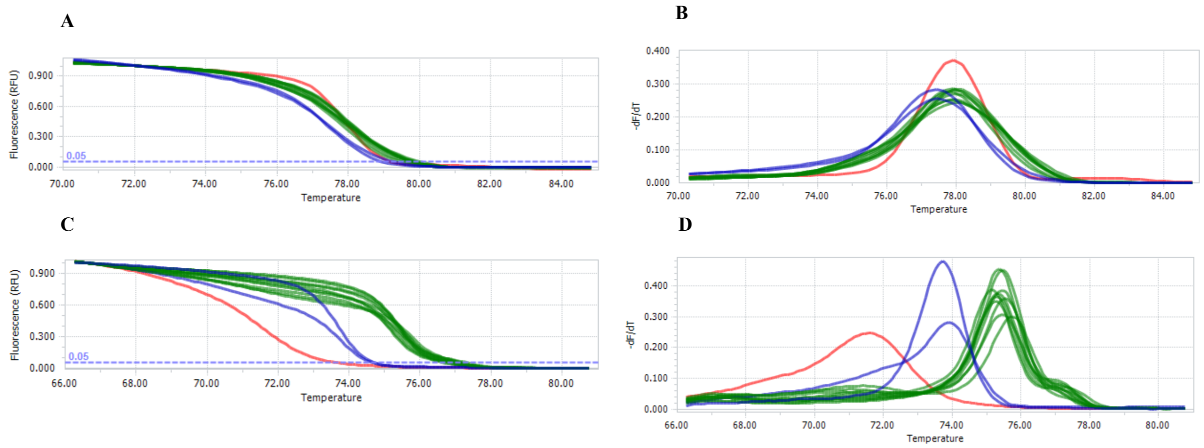

Supplementary Figure S4. PCR-HRM analysis of mosquitoes samples. A and **B**. Normalised melting curves and peaks using the Endmal18sF-R primers. **C** and **D**. Normalised melting curves and peaks using the Amzmal18sF-R primers. Blue curve: *P. falciparum*. Green curve: *P. vivax*. Red curve: *P. malariae*.

Supplementary Tables S2. Melting temperatures of the clinical and mosquito samples.

Melting temperatures of the clinical samples with the primers Endmal18sF-R						
**Sample ID**	**Blood smears**	**Snounou's nested PCR**	**PCR-HRM**	**Tm (°C)**		
				P. vivax	***P. malariae***	***P. falciparum***
HUMAN 1	+	+	POSITIVE			77.37
HUMAN 2	+	+	POSITIVE	77.66		
HUMAN 3	+	+	POSITIVE	77.94		
HUMAN 4	+	+	POSITIVE			77.44
HUMAN 5	+	+	POSITIVE	77.67		
HUMAN 6	-	-	POSITIVE		78.76	
HUMAN 7	-	-	POSITIVE		78.28	
HUMAN 8	-	-	POSITIVE	77.91		
HUMAN 9	-	-	POSITIVE	77.95		
HUMAN 10	+	+	POSITIVE			77.20
HUMAN 11	-	-	NEGATIVE			
HUMAN 12	-	-	NEGATIVE			
HUMAN 13	-	-	NEGATIVE			
HUMAN 14	-	-	NEGATIVE			
HUMAN 15	+	+	POSITIVE			77.50
HUMAN 16	+	+	POSITIVE			77.39
HUMAN 17	+	+	POSITIVE			77.44
HUMAN 18	+	+	POSITIVE			77.43
HUMAN 19	+	+	POSITIVE			77.38
HUMAN 20	+	+	POSITIVE			77.42
HUMAN 21	+	+	POSITIVE			77.52
HUMAN 22	+	+	POSITIVE			77.25
HUMAN 23	+	+	NEGATIVE			
HUMAN 24	+	+	POSITIVE			77.15
HUMAN 25	-	-	POSITIVE			77.46
HUMAN 26	-	-	NEGATIVE			
HUMAN 27	+	+	POSITIVE			77.47
HUMAN 28	+	+	POSITIVE	77.7		
HUMAN 29	+	+	POSITIVE	77.77		
HUMAN 30	+	+	POSITIVE	77.76		
HUMAN 31	+	+	POSITIVE	77.74		
HUMAN 32	-	+	NEGATIVE			
HUMAN 33	-	+	POSITIVE	77.72		
HUMAN 34	-	+	POSITIVE		78.23	
HUMAN 35	-	+	POSITIVE	77.89		
HUMAN 36	+	+	POSITIVE	77.68		
HUMAN 37	+	+	POSITIVE	77.8		
HUMAN 38	-	-	POSITIVE			77.27
HUMAN 39	-	-	POSITIVE			77.27
HUMAN 40	+	+	POSITIVE			77.16
HUMAN 41	+	+	POSITIVE	77.72		
			**AVERAGE**	**77.78**	**78.42**	**77.36**
			**Sd**	**0.15**	**0.34**	**0.11**

Melting temperatures of the clinical samples with the primers Amzmal18sF-R						
**Sample ID**	**Blood smears**	**Snounou's nested PCR**	**PCR-HRM**	**Tm (°C)**		
				P. vivax	***P. malariae***	***P. falciparum***
HUMAN 1	+	+	POSITIVE			73.67
HUMAN 2	+	+	POSITIVE	75.28		
HUMAN 3	+	+	POSITIVE	75.7	71	
HUMAN 4	+	+	POSITIVE			73.77
HUMAN 5	+	+	POSITIVE	75.4		
HUMAN 6	-	-	POSITIVE		71.08	
HUMAN 7	-	-	POSITIVE		71.05	
HUMAN 8	-	-	NEGATIVE			
HUMAN 9	-	-	NEGATIVE			
HUMAN 10	+	+	POSITIVE			73.77
HUMAN 11	-	-	POSITIVE			73.58
HUMAN 12	-	-	NEGATIVE			
HUMAN 13	-	-	NEGATIVE			
HUMAN 14	-	-	NEGATIVE			
HUMAN 15	+	+	POSITIVE			73.7
HUMAN 16	+	+	POSITIVE			73.68
HUMAN 17	+	+	POSITIVE			73.82
HUMAN 18	+	+	POSITIVE			73.79
HUMAN 19	+	+	POSITIVE			73.66
HUMAN 20	+	+	POSITIVE			73.5
HUMAN 21	+	+	POSITIVE			73.49
HUMAN 22	+	+	POSITIVE			73.49
HUMAN 23	+	+	POSITIVE			73.39
HUMAN 24	+	+	POSITIVE			73.49
HUMAN 25	-	-	NEGATIVE			
HUMAN 26	-	-	POSITIVE	75.3		
HUMAN 27	+	+	POSITIVE			73.87
HUMAN 28	+	+	POSITIVE	75.1		
HUMAN 29	+	+	POSITIVE	75.17		
HUMAN 30	+	+	POSITIVE	75.48		
HUMAN 31	+	+	POSITIVE	75.26		
HUMAN 32	-	+	POSITIVE	75.42		
HUMAN 33	-	+	POSITIVE	75.43		
HUMAN 34	-	+	POSITIVE	75.72	70.7	
HUMAN 35	-	+	POSITIVE	75.25		
HUMAN 36	+	+	POSITIVE	75.49		
HUMAN 37	+	+	POSITIVE	75.39		
HUMAN 38	-	-	NEGATIVE			
HUMAN 39	-	-	NEGATIVE			
HUMAN 40	+	+	POSITIVE			73.39
HUMAN 41	+	+	POSITIVE	75.67	71.1	
			**AVERAGE**	**75.40**	**70.99**	**73.64**
			**Sd**	**0.19**	**0.16**	**0.16**

Melting temperatures of mosquito samples with the primers Endmal18sF-R

Sample ID	Tm (°C)		
	P. vivax	*P. malariae*	*P. falciparum*
MOSQUITO 1	77.68		
MOSQUITO 2	77.86		
MOSQUITO 3	77.94		
MOSQUITO 4	77.91		
MOSQUITO 5	-		77.52
MOSQUITO 6	77.76		
MOSQUITO 7	77.97		
MOSQUITO 8	77.8		
MOSQUITO 9	77.88		
MOSQUITO 10	77.76		
MOSQUITO 11-342	NEGATIVE		
**AVERAGE**	**77.840**		**77.52**
**Sd**	**0.096**		

Melting temperatures of mosquito samples with the primers Amzmal18sF-R

Sample ID	Tm (°C)		
	P. vivax	*P. malariae*	*P. falciparum*
MOSQUITO 1	75.14		
MOSQUITO 2	75.6		
MOSQUITO 3	75.74		
MOSQUITO 4	75.42		
MOSQUITO 5	NEGATIVE		73.82
MOSQUITO 6	75.5		
MOSQUITO 7	75.49		
MOSQUITO 8	75.28		
MOSQUITO 9	75.63		
MOSQUITO 10	75.43		
MOSQUITO 11-342	NEGATIVE		
**AVERAGE**	**75.47**		**73.82**
**Sd**	**0.182**		

Supplementary Table S3. Theoretical melting temperatures of *P. brasilianum* and *P. simium*. The theoretical melting temperatures (T_m) were calculated using OligoCalc (http://biotools.nubic.northwestern.edu/OligoCalc.html) and uMelt v2.0.2 (http://www.dna. utah.edu/umelt/um.php), for *P. brasilianum* and *P. simium*. The GenBank accession numbers listed were used to simulation of theoretical temperature. The sequences were aligned in Mega 6. Only the sequences with the full amplicons were analysed.

***Plasmodium* species**	**GenBank accession**	**Amzmal18SF-R theoretical T_m**	
		**uMelt**	**OligoCalc**
P. brasilianum	KC906728	71.4	74.25
P. simium	U69605	76.7	76.8
